# Immune Tolerance of the Human Decidua

**DOI:** 10.3390/jcm10020351

**Published:** 2021-01-18

**Authors:** Hiromi Murata, Susumu Tanaka, Hidetaka Okada

**Affiliations:** 1 Department of Obstetrics and Gynecology, Kansai Medical University, 2-5-1 Shinmachi, Hirakata, Osaka 573-1010, Japan; murathir@hirakata.kmu.ac.jp; 2 Department of Anatomy, Kansai Medical University, 2-5-1 Shinmachi, Hirakata, Osaka 573-1010, Japan

**Keywords:** endometrium, immune tolerance, endometrial stromal cells, uterine natural killer cells, regulatory T cells (Treg), macrophages, heart- and neural crest derivatives-expressed protein 2 (HAND2), interleukin-15 (IL15), galectin 9

## Abstract

The endometrium is necessary for implantation, complete development of the placenta, and a successful pregnancy. The endometrium undergoes repeated cycles of proliferation, decidualization (differentiation), and shedding during each menstrual cycle. The endometrium—including stromal, epithelial, vascular endothelial, and immune cells—is both functionally and morphologically altered in response to progesterone, causing changes in the number and types of immune cells. Immune cells make up half of the total number of endometrial cells during implantation and menstruation. Surprisingly, immune tolerant cells in the endometrium (uterine natural killer cells, T cells, and macrophages) have two conflicting functions: to protect the body by eliminating pathogenic microorganisms and other pathogens and to foster immunological change to tolerate the embryo during pregnancy. One of the key molecules involved in this control is the cytokine interleukin-15 (IL-15), which is secreted by endometrial stromal cells. Recently, it has been reported that IL-15 is directly regulated by the transcription factor heart- and neural crest derivatives-expressed protein 2 in endometrial stromal cells. In this review, we outline the significance of the endometrium and immune cell population during menstruation and early pregnancy and describe the factors involved in immune tolerance and their involvement in the establishment and maintenance of pregnancy.

## 1. Introduction

The human menstrual cycle duration ranges from 24–38 days [1]. During the reproductive age of today’s women, the cycle occurs nearly 450 times until a woman reaches menopause [1,2,3]. The human endometrial lining undergoes regeneration, differentiation, and shedding during each menstrual cycle. Therefore, the endometrial cycle is divided into three dominant phases: the proliferative phase, the secretory phase, and the menstrual phase. These phases are governed by the changes of two ovarian steroid hormones, estrogen (E2) and progesterone (P4). The endometrium, including stromal, epithelial, vascular endothelial, and immune cells, is both functionally and morphologically altered in response to these hormonal levels [4]. In the proliferative phase, the endometrium regenerates and proliferates under the influence of increased E2 levels due to the growth of ovarian follicles. After ovulation, the development of the corpus luteum occurs, starting with the ruptured follicle, and P4 is secreted from the corpus luteum. In the secretory phase, endometrial glandular epithelial cells transform into a secretory form and endometrial stromal cells (ESCs) differentiate into decidual cells in response to increasing P4 [5]. Menstruation is triggered by a decrease in P4 levels due to the absence of implantation and reset of the endometrial cycle occurs. Once implantation is established, syncytiotrophoblasts increasingly produce human chronic gonadotropin to preserve P4 levels by maintaining the corpus luteum [6]. The production of P4 by the corpus luteum is essential for supporting embryo implantation and the establishment of the placenta [7].

## 2. Decidualization: Morphological Differentiation in the Human Endometrium

**Figure 1:**
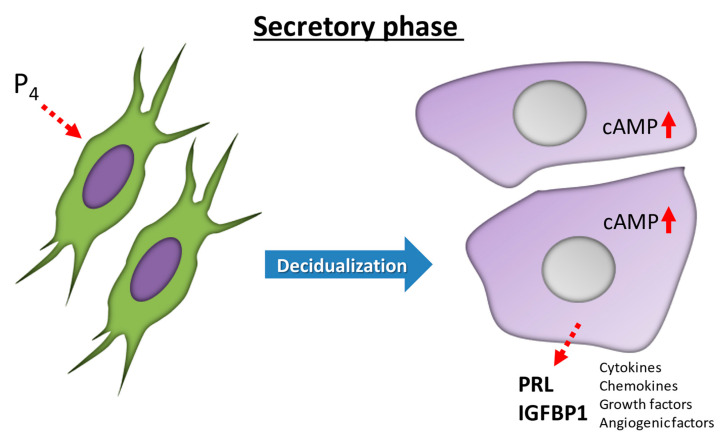
**Differentiation in human endometrial stromal cells (ESCs) during decidualization.** Decidualization is driven by increases in progesterone (P4) and then local cyclic adenosine monophosphate (cAMP) production. In the human endometrium, ESCs transform from fibroblast-like cells in the proliferative phase to epithelium-like cells with cytoplasmic expansion, large pale nuclei, and rounded shapes in the secretory phase. IGFBP-1, insulin-like growth factor binding protein-1; PRL, prolactin.

Decidualization is characterized by significant functional and morphological differentiation of human ESCs, and is critical for blastocyst implantation and the maintenance of pregnancy [2]. Decidualization is driven by increases in P4 and then local cyclic adenosine monophosphate (cAMP) production [8,9]. In the human endometrium after ovulation, ESCs transform from fibroblast-like cells in the proliferative phase to epithelium-like cells with cytoplasmic expansion, large pale nuclei, and rounded shapes in the secretory phase [10] (Figure 1), a process that involves complex cytoskeletal rearrangements [11]. The phosphorylation of myosin light chain and concentrated F-actin induce the intracellular remodeling and resulting morphological changes [12,13,14]. These morphological changes in human ESCs are observed even during in vitro decidualization by P4 or cAMP stimulation [9,15,16,17,18]. P4 and local cAMP production enhance the biosynthesis of intracellular complex networks and secreted proteins necessary for decidualization [19]. In presence of P4, ESCs also transform into epithelium-like forms and secrete decidual proteins, such as insulin-like growth factor binding protein-1 (IGFBP-1) and prolactin (PRL) [17,18] (Figure 1). P4 also enhances the production of several factors, including interleukin-15 (IL-15) [20,21,22,23,24,25]. The P4 receptor antagonist, RU-486, completely inhibits P4-induced PRL production during decidualization [15].

## 3. Functional Differentiation in Human Endometrium: IGFBP-1 and PRL as Decidual Markers

P4 functions by binding to and activating the progesterone receptor (PGR) [26]. Ligand-binding PGR is recruited to P4-response elements in the promoters of target genes and regulates their transcription [27]. PGR pathways and/or accumulations of cAMP induce the expression of decidual transcriptional regulators, epigenetic modifications, rearrangement of signal transductions, and posttranscriptional modifications [8]. Once the decidualization begins, decidual ESCs secrete a number of cytokines, chemokines, growth factors, and angiogenic factors to promote decidualization for blastocyst implantation (Figure 1). The decidual process proceeds by interacting with all cells in the endometrium, including decidual ESCs, glandular cells, vascular endothelial cells, and local immune cells [28]; consequently, decidual ESCs secrete many specific proteins including IGFBP-1 and PRL. IGFBP-1 and PRL stimulate trophoblast growth and invasion via the PRL receptor and/or integrins [29,30]. 

The decidua, cytotrophoblasts, placental trophoblasts, and amniotic epithelial cells express PRL receptors [31]. Moreover, endometrial stromal and glandular cells exhibit PRL receptor expression during the menstrual cycle [32]. It has been suggested that PRL may play a role in the implantation process through immune environment modification and/or regulation of the factors that control trophoblast proliferation and invasion into the endometrium [33,34]. Upregulation of functional PRL receptors is found in the secretory phase of ESCs and glandular cells [35]. Further, decidual PRL may influence glandular epithelial function/secretion through a paracrine mechanism and direct gene transcription via the Janus kinase/signal transducer and activator of transcription signaling [34,36].

An increase in cell migration is the main cause of trophoblast invasion [37,38]. Trophoblast invasion of the human uterus is mediated through cell surface integrins. IGFBP-1 stimulates cell migration; it has been substantiated that in vitro trophoblast migration needs integrin α5 and β1 subunits [37]. Moreover, IGFBP-1 has the potential to induce ESC decidualization via integrin α5β1 [30].

## 4. Spontaneous Decidualization of Human ESCs

**Figure 2:**
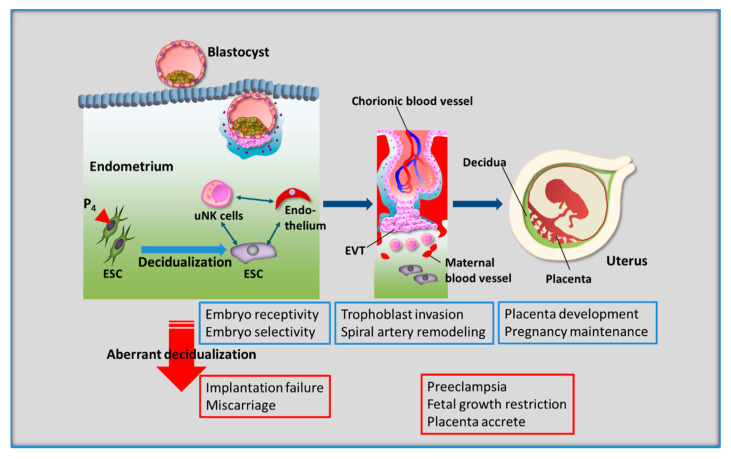
**Decidualization of human endometrial stromal cells (ESCs) is essential for reproductive success.** During the secretory phase, decidualized ESCs interact with all cells in the endometrium, including uterine natural killer (uNK) cells, which are representative local immune cells, vascular endothelial cells, and blastocysts. This decidual process leads to successful pregnancy, i.e., embryo implantation, balanced trophoblast invasion, spiral artery remodeling, establishment of the placenta, and the maintenance of pregnancy. Therefore, aberrant decidualization results in reproductive and perinatal impairment, including implantation failure, miscarriage, preeclampsia, fetal growth restriction, and placenta accreta. EVT, extravillous trophoblast cell.

In contrast to several most other mammals, the spontaneous decidualization of human ESCs occurs even without blastocyst implantation. The occurrence of decidualization independent of the presence of a blastocyst is observed in a handful of species, including some primates (humans, apes, and Old World monkeys), some bats, spiny mice, and the elephant shrew [8,39,40,41,42,43]. In a recent study, the ancestral gene regulatory program from which the core network of decidual ESCs evolved has been identified due to analyzing in vitro response of opossum endometrial stromal fibroblasts (ESFs) to progesterone and cAMP which differentiate human ESFs into human decidual ESCs [44]. As core components of the decidual gene regulatory network are responsive to stimuli in opossum ESF, components of cellular stress responses, such as apoptotic and oxidative stress response, rather than undergoing human ESC differentiation were determined. This opossum study suggests that the decidual ESCs evolved based on a physiological stress response that appears to be directly concerned with the invasion of trophoblast into maternal endometrium [44]. There is a high prevalence of chromosomally abnormal preimplantation embryos in humans, therefore reproductive success is largely limited [45,46]. Human ESCs are suggested to be potential biosensors for embryo quality upon decidualization [47]. It is believed that the human endometrium is essentially capable of adaption to variations in embryo quality by rebalancing its receptivity and selectivity traits [48]. Previous studies showed that decidualized ESCs act as both a gatekeeper as well as a chief modulator against local immune cells [48]. Human decidualized ESCs selectively recognize developmentally impaired human embryos and inhibit secretion of key implantation mediators (e.g., IL-1β and heparin binding epidermal growth factor) and immunomodulators (e.g., IL-5, -6, -10, -11, -17, and eotaxin), whereas undifferentiated ESCs fail to recognize them [47]. Mid-secretory endometrial biopsies from 10 women with recurrent pregnancy loss showed decreased *PRL* mRNA [49]. Further, differentiated ESCs from women with recurrent pregnancy loss demonstrate attenuation in *PRL* mRNA [49]. Furthermore, ESCs from women with recurrent miscarriage have a higher migratory response to trophoblast spheroids than ESCs from normally fertile women [50]. Increasing evidence suggests that impaired decidualization predisposes to late implantation, causes quality control malfunction of embryo development, and induces early placental insufficiency, regardless of the embryonic karyotype; thus, recurrent pregnancy loss is likely to be the result of these processes. In other words, spontaneous decidualization is not only necessary for the development of placenta, but also for the ability to perceive, respond to, and eliminate the implantation of defective embryos [51] (Figure 2).

## 5. Uterine Natural Killer (uNK) Cells in Human Endometrium

The essential roles of decidualization are to avoid the embryo from maternal immunological refusal and to provide a nutritional environment for the developing embryo before placentation [52]. The major secretory components from ESCs, PRL and IGFBP-1, not only stimulate trophoblast growth, but also prevent maternal immunological rejection, modulate local immune cells, including uNK cell survival, and promote angiogenesis [8,16,53]. uNK cells are the most prominent immune cells in the endometrium [54] and make up ~70% of all white blood cells in the human endometrium during the secretory phase and early pregnancy [55,56]. In contrast to peripheral NK cells that are predominantly CD56^dim^, CD16^+^, uNK cells are mainly CD56^bright^, CD16^-^ and are poorly cytotoxic lymphocytes [56]. uNK cells have important roles in the establishment and maintenance of early pregnancy, such as promotion of angiogenesis in decidua, remodeling of spiral arteries, and trophoblast invasion [56,57,58]. Recent studies indicate that human uNK cells co-operating with decidual cells eliminate senescent decidual cells which resists P4 and pro-senescent decidula response associates with recurrent pregnancy loss [48,59,60]. These results provide new insights into the pivotal role of innate immune cells in preventing the destruction in human endometrium caused by excessive senescence occurring in early pregnancy.

Generally, the function of NK cell is controlled via their membrane NK cell receptors that bind to major histocompatibility complex (MHC) class I molecules and non-MHC ligands [61]. After implantation, placental extravillous trophoblast cells (EVTs) invade the decidua and migrate towards the spiral artery [62]. Although sufficient changes of the arteries are required, excessive invasion must be prevented to ensure appropriate allocation of resources to the mother and baby [63]. Hence, the invasion of EVTs needs to be properly controlled. In cases with placenta accreta where the placenta implants on a previous Caesarean section scar, i.e., in the absence of decidua, uncontrolled and life-threatening trophoblast invasion occurs, thus identifying the pivotal role of the decidua [64]. Fetal EVTs have a unique human leukocyte antigen (HLA) profile: they do not have class I HLA-A and HLA-B, or class II molecules, which are dominant T cell ligands [65,66], but do have polymorphic HLA-C class I molecules, HLA-E, and HLA-G [67]. Disturbance of antigen presentation on EVTs is induced based on the absence of these HLA molecules [67]; therefore, their absence facilitates one of the key mechanisms to avoid T-cell recognition of invading fetal cells. Moreover, EVT HLA ligands interact with NK cell receptors that are expressed on uNK cells [28]. 

**Figure 3:**
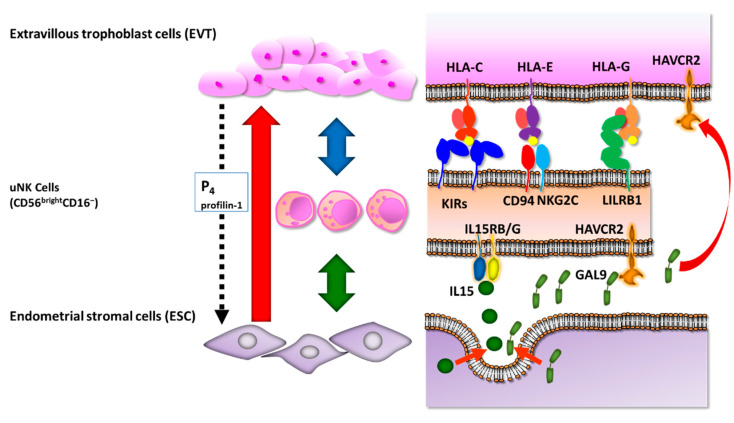
Molecular interactions among extravillous trophoblast cells, uterine natural killer (uNK) cells, and endometrial stromal cells. Extravillous trophoblast cells express human leukocyte antigen (HLA)-C, HLA-G, and HLA-E class I molecules and interact with killer cell immunoglobulin-like receptors (KIRs), activating C-type lectin receptor CD94/NKG2C, and leukocyte immunoglobulin-like receptor, subfamily B (LILRB1) on the surface of uNK cells to avoid immunological recognition. Endometrial stromal cells secrete interleukin (IL)-15 and galectin-9 (GAL9) to suppress inflammatory reactions of uNK cells via IL-15RB/G and Hepatitis A virus cellular receptor 2 (HAVCR2). EVT secretes progesterone (P4) and profilin-1 to regulate ESC decidualization.

For instance, uNK cells express killer cell immunoglobulin-like receptors (KIRs), including inhibitory KIR2DL1, KIR2DL2, and KIR2DL3 receptors, in addition to activating KIR2DS1 and KIR2DL4 receptors, some of which bind to HLA-C molecule [68,69,70] (Figure 3). Allorecognition of paternal HLA-C by maternal KIRs may influence trophoblast invasion and vascular remodeling, with subsequent effects on placental development and pregnancy outcome [71,72]. Pregnancy disorders, including recurrent pregnancy loss, pre-eclampsia, and fetal growth restriction share a common primary pathogenesis of defective arterial transformation, assisted by the same combination of maternal KIRs and fetal HLA-C genotypes [68,73]. A combination of “a paternally derived HLA-C2 epitope” and “increased frequency of KIR AA genotype in mother” is associated with the pregnancy disorders [71].

It is well known that HLA-E binds to C-type lectin receptor CD94/NKG2 heterodimers, inhibitory NKG2A, and activating NKG2C [74,75]. CD94/NKG2A is highly prevalent on the uNK cells with strong expression [76] (Figure 3). As a result, the total CD94/NKG2 interaction with HLA-E inhibits the cytotoxic effects of decidual NK cells [76]. 

HLA-G binds to members of the leukocyte immunoglobulin-like receptor, subfamily B (LILRB) family, including LILRB1 and LILRB2 [77]. LILRB1 functions as an inhibitory receptor for peripheral blood NK cells, whereas it acts as an activating receptor in the decidua [78]. The LILRB1 receptor is found on approximately 30%–40% of uNK cells. HLA-G binding to LILRB1/2 on responding antigen-presenting cells (APCs) inhibits the proliferation of allogeneic lymphocytes [79]. HLA-G is the only HLA-I molecule that forms dimers with β2-microglobulin to increase avidity against LILRB1 in the endometrium. Thus, decidual APCs are suppressed by a placental-specific signal from an HLA-G-LILRB1/2 interaction [79] (Figure 3). 

Cultured EVTs from human chorionic villi explants secrete progesterone [80]. In vitro EVT secretes profilin-1, which acts to promote ESC decidualization via the down-regulation of ALOX5 in ESC [81]. Profilin-1 also down-regulates ALOX5 in macrophages where it likely regulates cytokine production and induces immune tolerance [81] (Figure 3).

## 6. IL-15

**Figure 4:**
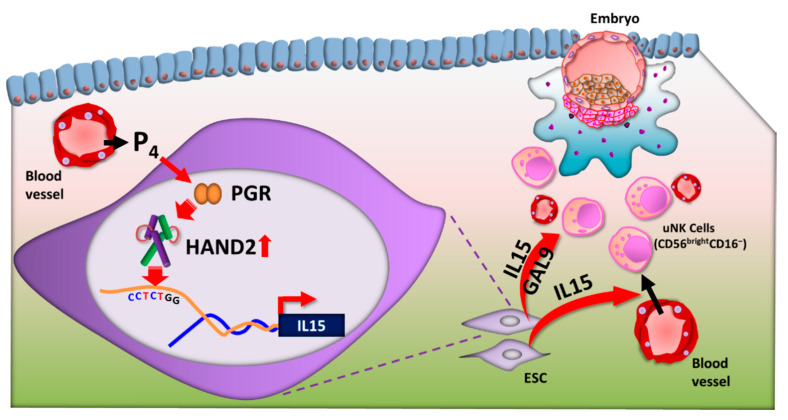
**Regulation of uterine natural killer (uNK) cells by heart- and neural crest derivatives-expressed protein 2 (HAND2).** After ovulation, progesterone (P4) is secreted from the corpus luteum. In response to increasing P4, endometrial stromal cells (ESCs) differentiate to decidual cells in the secretory phase. P4-binding progesterone receptor (PGR) is recruited to P4-response elements in the promoters of *HAND2* and upregulates HAND2 expression. HAND2 directly upregulates *IL-15* transcription via the HAND2 motif (CCTCTGG) in the upstream region of the *IL-15* gene in ESCs. IL-15 is secreted from ESCs and has a critical role in regulation of the differentiation and function of uNK cells. GAL9, galectin 9.

As uNK cells arise from maternal endometrial progenitors, their repertoires form in response to local signals from fetal EVTs as well as endometrial immune, epithelial, glandular, and stromal cells in human [56,71]. IL-15 that is secreted in the secretory phase plays a main role in postovulatory restitution of peripheral blood NK cells into the human endometrial tissues [82] (Figure 4). IL-15-deficient mice are depleted for uNK cells, indicating that uNK cells require IL-15 for their development [83]. Moreover, incubation in decidual ESC conditioned medium supplemented with IL-15 and stem cell factors converts peripheral blood NK cells to cells that phenotypically resemble decidual uNK cells in human [84]. Moreover, uNK cells are activated by IL-15 secreted from differentiated human decidual cells [59]. Then, activated uNK cells eliminate senescent endometrial cells via exocytosis of cytotoxic granules [59]. However, it is unclear whether uNK cells originate from endometrial precursors or are replenished from peripheral NK cells. Although IL-15 is essential for uNK cell differentiation and is secreted from ESCs via P4 stimulation, uNK cells and other endometrial white blood cells do not express the P4 receptor in human [85].

IL-15 is a 14–15 kDa polypeptide and a member of the 4-α-helix bundle cytokine family [86]. Generally, the intracellular effects of IL-15 are mediated via a heterotrimetric membrane receptor comprising IL-2RB, IL-2RG, and IL-15RA [87]. Several studies indicate that IL-15 plays various important roles in NK cell biology via binding these receptors [88]. Previous quantitative polymerase chain reaction (PCR) studies show that *IL15* expression is significantly higher during the secretory phase in the human endometrium [10,25]. Histological analysis using human endometrial tissues also indicates that *IL15* increases in the secretory phase [10] and is observed in many ESCs in the endometrium during this phase [10]. Moreover, cultured human ESCs increase IL-15 secretion during the progestin-induced decidualization [89]. A customized microarray (endometrial receptivity array) has defined *IL15* as a significant indicator of the endometrial window of implantation [90]. Recent single cell analysis from human first-trimester placentas further suggests that the interaction between IL-15 and a heterodimetric receptor IL-15RB/G is one of the critical events between decidual stromal cells and decidual NK cells [28] (Figure 3). Taken together, these observations indicate that IL-15 produced by ESCs has a critical role in regulation of the differentiation and function of uNK cells in human endometrium. 

## 7. Heart- and Neural Crest Derivatives-Expressed Protein 2 (HAND2): A key Decidua Transcription Factor for *IL15* Transcription

To date, although some decidua transcription factors have been reported, such as PGR [91,92], homeobox A10 [93,94], forkhead box O1 (FOXO1) [95,96], the STAT families, and HAND2 [97,98], in both humans and animals, transcription factors that directly affect *IL15* transcription in human ESCs have not been identified. However, a recent study confirmed that HAND2 directly upregulates human *IL15* transcription in ESCs [10] (Figure 4).

In murine fetal development, HAND2 was originally identified as a transcription factor required for embryonic heart development [99,100]. Further, in the reproductive field, HAND2 has a crucial role in the receptivity and implantation of embryos in mice [97,101]. During decidualization, *HAND2* expression in human ESCs is altered by medroxyprogesterone, a representative progestin, in a dose- and time-dependent manner [102]. PGR recruits to the promoter region of the *HAND2* locus and PGR knockdown induces differential gene expression during decidualization in human [103]. Furthermore, the PGR antagonist, RU486, blocks the induction of *HAND2* mRNA expression in human ESCs [102,104]. Therefore, these data suggest that PGR directly regulates *HAND2* transcription. 

*HAND2* expression is also significantly increased during the secretory phase in the human endometrium, as determined by quantitative PCR and histological analysis [10]. Moreover, *HAND2* and *IL15* transcription in the human endometrium showed strong positive correlation during the menstrual cycle [10]. In human ESCs, *HAND2* silencing reduces both morphological differentiation and decidual-specific factors, including *PRL*, fibulin-1, *FOXO1A*, tissue inhibitor of metalloproteinase-3, and *IL15* [98]. Hence, HAND2 is a master mediator of P4 action in human ESC decidualization. A recent study identified a CCTCTGG sequence as a HAND2 motif in the upstream (promoter) region of the human *IL15* gene; HAND2 directly upregulates *IL15* transcription in ESCs through this motif [10] (Figure 4). 

## 8. T Cell

CD4 + CD25 + FOXP3 + regulatory T cells (Tregs), which are formerly known as suppressor T cells, and a subpopulation of T cells that modulate the immune system, maintain tolerance to self-antigens, prevent autoimmune disease, and increase in the decidua at implantation site and in early pregnancy until mid-gestation of human [105]. Tregs were originally identified in mice as immunosuppressive and generally suppress or downregulate induction and proliferation of effector T cells [106]. Tregs are important in mediating maternal immune tolerance to the allogeneic fetus during embryo implantation and early pregnancy, but may not be necessary for maintenance of the late allogeneic pregnancy in mammals [107,108,109,110,111]. Treg cells are critical for maternal tolerance of the embryo, embryolemma, and placenta in mice, and the Treg cell pool expansion via antigen-specific and nonspecific pathways allows their suppressive effects to be exerted during the critical peri-implantation phase of pregnancy [112]. In human beings, the accumulation of Treg cells in the decidua and the elevation in maternal blood are found from early in the first trimester [112]. In women, insufficient numbers of Treg cells or their functional deficiency have been linked to infertility, miscarriage, and preeclampsia [112]. Studies conducted in animal models have shown that depletion of Treg cells leads to the greatest elevation in miscarriage rates, which were associated with the expansions of activated CD4+ and CD8+ T cells occurring only in the uterine draining lymph nodes [113]. Women with recurrent pregnancy loss have decreased Treg cells even in their peripheral blood compared to normal women [105,114]. In humans, the incidence of repeated spontaneous abortion (RSA) elevates with decreased or increased levels of Treg or Th17 cells, respectively [115]. A higher Th17/Treg cell ratio at the fetal-maternal interface was observed in a woman with an unknown RSA history [116].

Although no change in the CD8^+^ cell number was found in the human endometrium, endometrial CD8^+^ T cytotoxicity is maintained during the proliferative phase but that activity is suppressed by the decidua microenvironment during the secretory phase [28,117,118]. There is a general agreement that pregnancy is associated with Th2 dominance, and Th1 immune response is associated with embryonic rejection in human [119]. Intracellular Th1 cytokine expressions are increased over Th2 cytokine expressions in women with RSA and infertility of multiple implantation failures [120]. 

**Figure 5:**
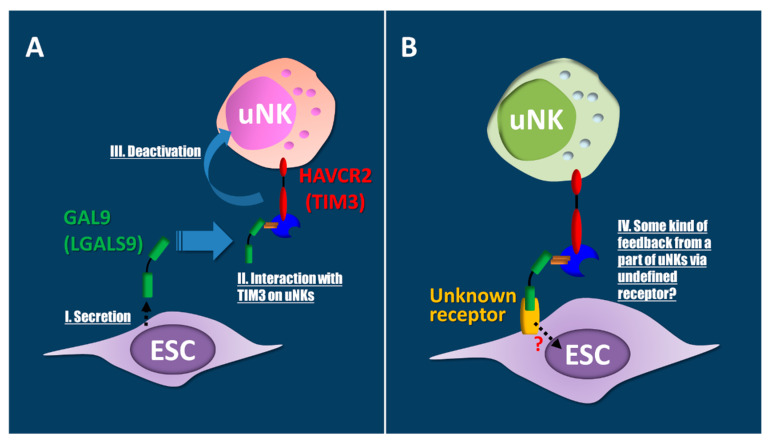
**Endometrial stromal cells (ESCs) may be regulated by a Hepatitis A virus cellular receptor 2 (HAVCR2)-galectin 9 (GAL9) interaction via unknown receptors on ESCs.** (**A**) Decidual ESCs increase secretion of the ligand GAL9 (I). Secreted GAL9 interacts with HAVCR2 on uterine natural killer (uNK) cells (II). These interactions induce the deactivation of uNKs (III). As a result, immune tolerance, apoptosis, and eliminations may occur, similar to that in Th1-macrophage interactions. (**B**) Feedback regulations of the HAVCR2-GAL9 interaction via known or unknown membrane proteins are suggested in ESCs through uNKs (IV).

A recent report showed that the interaction between all cytotoxic maternal T or NK cells and fetal trophoblast cells are blocked in the human decidua microenvironment [28]. In humans, specifically, high levels of PDL1, a ligand for PD1, that suppresses the damaging cells was found in EVTs, which first invade the decidual ESCs with high galectin 9 (GAL9; also called LGALS9) expression [28]. GAL9, secreted by human ESCs, interacts with their respective inhibitory receptors and Hepatitis A virus cellular receptor 2 (HAVCR2), which is expressed by subsets of uNKs [28], thereby enabling decidual ESCs to suppress inflammatory responses (Figure 3). In mice, HAVCR2, a newly defined regulatory factor, downregulates T helper (Th)1 responses through transduction of apoptosis signaling by engaging GAL9 [121,122]. Thus, HAVCR2 may regulate the Th1-Th2 balance even in the human endometrium. The percentage of uNK cells with HAVCR2 expression is decreased in human miscarriages and abortion-prone murine models [123]. Moreover, decreased Th2-cytokine and increased Th1-cytokine levels are observed in uNK cells with HAVCR2 expression, but not in those without HAVCR2 expression from human and murine miscarriages [123]. Hence, the decidual immunological microenvironments could potentially suppress inflammatory reactions that are induced by trophoblast invasion. It has been suggested that macrophages that secrete GAL9 ligand are activated by Th1 cells expressing HAVCR2 through HAVCR2-GAL9 interactions with an unidentified GAL9 receptor on the macrophage cell surface in mice [124]. Therefore, the next research step using human samples should focus on experiments with addition of HAVCR2 peptides to the medium or HAVCR2-coated culture dishes to elucidate HAVCR2-GAL9 interactions between uNK and ESCs (Figure 5).

## 9. Other Immune Cells

Several other immune cells exist in the human superficial endometrial layer and mature gradually from the proliferative to ovulatory phase during the menstrual cycle. In addition to the predominant uNKs and T cells, macrophages, mast cells, neutrophils, dendritic cells (DCs), and B cells are also present in the human endometrium and may participate in immune tolerance and embryo implantation [125]. In women, the percentage of endometrial immune cells varies according to the phases of menstrual cycle [125]. Immune cells make up 30% of the total number of cells in the human endometrium during early pregnancy [126,127]. Although uNKs and other endometrial white blood cells apparently respond to P4, these leukocytes do not express PGR [85]. P4 exerts its effects through ESCs that express PGRs and may indirectly communicate with immune cells via ESC-secreted soluble factors, including cytokines in human.

In women, although a very low number of CD45RA^+^ B cells is found all the time during the cycle [118], whether or not these cells produce immunoglobulin within the tissue has not been identified yet.

CD68^+^ macrophages are also found during all phases of the menstrual cycle with increased numbers during the proliferative phase in human [118,128]. They are found scattered all over the human endometrium, especially around the glands [129]. The number of these macrophage is significantly increased prior to menses during the secretory phase, and a notable increase is found at the implantation site in human [130,131]. The density of human endometrial mature CD83^+^ DCs is significantly lower than that of immature CD1a^+^ DCs [130]. However, there is no difference in the number of CD1a^+^ and CD83^+^ DCs in the fundus and isthmus of the human uterus [130]. During implantation and subsequent pregnancy in mice, both macrophages and DCs conglomerate around the decidua and in the uterus [132]. Then, uterine DCs start to produce transforming growth factor (TGF)-β1 [133], which promotes Treg cells and suppresses cytolytic CD8^+^ T cells in mice [134]. These studies suggest that successful decidualization and embryo implantation need endometrial DCs in animals. Even in humans, the proportion of DC1 cells is increased compared to that of DC2 cells [28]. Additionally, co-expression of programmed cell death protein 1 (PD1) has also been found [28], suggesting that the functions of decidual CD8^+^ T cells might be suppressed by DC1 cells in human. PD1 regulates immune responses as an immune-checkpoint protein [135,136,137,138]. Therefore, it is suggested that local T-cell activation in the human endometrium is limited by uterine DCs [28]. On the other hand, the differentiation of Treg cells are induced by tolerogenic DCs having immunosuppressive properties in the endometrium and other tissues in human [139,140,141]. Additionally, human endometrial mast cells are perpetually found during the menstrual cycle, and that mast cell activation is most pronounced immediately before menstruation [142].

Neutrophils have been detected in the human endometrium based on the presence of the neutrophil-specific protease elastase and their morphology [143]. CD11b^bright^, CD66b^+^, and CD16^+^ cells in the human endometrium are defined as endometrial neutrophils [144]. Neutrophils are almost undetectable in the normal human endometrium; however, their proportion rapidly reaches 6%–15% during the perimenstrual stage [143]. In patients with high-dose oral progestin administration, increased neutrophils are also found in the endometrial breakdown areas [145]; in patients with implanted levonorgestrel, they reached densities similar to those seen in the menstrual endometrium [146]. Eosinophils have also been detected with eosinophil cationic proteins in the human endometrium [143]. Like neutrophils, eosinophils are absent in the normal human endometrium during most of the menstrual cycle, but drastic and immediate increase in their number occurs prior to menstruation [142,146].

## 10. Conclusions

In this paper, we investigated immune tolerance in human endometrium and decidua mainly during implantation and early pregnancy. The role of uNK cells differentiation and activation promoted by decidual ESCs secreted-IL15, and the essential role of Treg cells in the decidua at implantation site from early pregnancy to mid-gestation are becoming more and more obvious.

Meanwhile, HAND2 acts as a master mediator of P4 action in decidualization for ESCs. Further investigations into the factors regulated by HAND2, HAND2 post-translational modifications, and their interactions will help understand the pathogenesis of immune tolerance in the endometrium. It is expected that these studies will ultimately lead to the elucidation of the mechanisms of implantation failure and embryo miscarriage with unknown origin and result in therapeutic development.

## Data Availability

No new data were created or analyzed in this study. Data sharing is not applicable to this article.
